# The *in vitro* inertial positions and viability of cells in suspension under different *in vivo* flow conditions

**DOI:** 10.1038/s41598-020-58161-w

**Published:** 2020-02-03

**Authors:** Sinead Connolly, Kieran McGourty, David Newport

**Affiliations:** 1 School of Engineering, Bernal Institute, University of Limerick, Limerick, Ireland; 2 School of Natural Sciences, Bernal Institute, University of Limerick, Limerick, Ireland

**Keywords:** Biomedical engineering, Fluid dynamics, Lab-on-a-chip, Biophysics

## Abstract

The influence of Poiseuille flow on cell viability has applications in the areas of cancer metastasis, lab-on-a-chip devices and flow cytometry. Indeed, retaining cell viability is important in the emerging field of cell therapy as cells need to be returned to patients’ bodies. Despite this, it is unclear how this fundamental fluid regime affects cell viability. This study investigated the influence that varying flow rate, and the corresponding wall shear stress (*τ*_*w*_) has on the viability and inertial positions of circulating cells in laminar pipe flow. The viability of two representative cell lines under different shear stresses in two different systems were investigated while particle streak imaging was used to determine their inertial positions. It was found that peristaltic pumps have a negative effect on cell viability in comparison to syringe pumps. Increasing shear stress in a cone and plate above 3 Pa caused an increase in cell death, however, *τ*_*w*_ as high as 10 Pa in circulation has little to no effect on cell viability. Inertial lift forces that move cells towards the centre of the channel protect them from experiencing detrimental levels of *τ*_*w*_, indicating that *τ*_*w*_ in Poiseuille flow is not a good predictor of cell viability during advection.

## Introduction

When cells are transported in suspension at varying concentrations in channels, they can experience a wide range of flow induced shear that is a complex function of the cell’s position, shape and the local flow distribution about the cell. *In vivo*, red blood cells, lymphocytes and cancer cells advect through both the cardiovascular system (CS) and lymphatic system (LS). *In vitro* systems, such as lab-on-a-chip devices, flow cytometers and cell therapy systems, also transport living cells at high velocities and in the case of cell therapy, re-introduce the processed cells back into the body. A key concern for the latter is that the fluid processing does not compromise the viability of the advecting cells.

**Figure 1:**
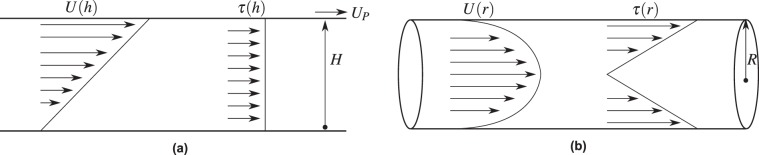
The velocity (*U*) and shear stress ($\tau$) profiles of (**a**) Couette flow and (**b**) Poiseuille flow where *U*_*P*_ is the velocity of the upper plate (the lower plate is stationary), *H* is the distance between the two plates and *R* is the radius of the pipe.

Whilst the local shear distribution around the cell represents the fluidic context, it is extremely challenging to quantify from a measurement or simulation perspective. A preferential approach is to use the maximum wall shear stress, the shear stress distribution or shear stress gradient in the region occupied by the cell, assuming the cell imparts a small perturbation on the local flow in that region. Several studies to date have examined the effects of fluid shear on cell viability utilizing different methods, with contrasting results. These studies generally replicate either Couette or Poiseuille flow in order to expose cells to a fixed shear value. Couette flow, is the laminar, shear-driven flow of a fluid between two plates of infinite depth, one of which is stationary while the other is in motion. This produces a linear velocity profile ($U(h)$), with respect to the vertical position in between the plates, *h*, as can be seen in Fig. 1a and is defined as $U(h)=\frac{{U}_{P}h}{H}$ where ${U}_{P}$ is the velocity of the upper plate and $H$ is the distance between the two plates. Due to the no-slip condition, $U$ is at a maximum at the moving plate, and equal to 0 at the stationary plate. A constant shear stress ($\tau (h)$) is produced between the two plates (also visible in Fig. 1a), $\tau (h)=\mu \frac{dU(h)}{dh}=\frac{\mu {U}_{P}}{H}$ where $\mu$ is the fluid viscosity. Poiseuille flow is the fully developed, laminar, pressure-driven flow of an incompressible fluid in a circular pipe. The velocity profile, $U(r)$, with respect to the channel radial position, *r*, of Poiseuille flow can be seen in Fig. 1b and can be described using the equation $U(r)=2\bar{U}(1-\frac{{r}^{2}}{{R}^{2}})$ where $\bar{U}$ is the mean fluid velocity and *R* is the channel radius. This results in a fluid velocity of 0 at the channel wall with the maximum fluid velocity at the channel centre. The shear stress distribution across the channel width, $\tau (r)=\mu \frac{dU(r)}{dr}=-\,\frac{4\mu \bar{U}r}{{R}^{2}}$. As can be seen from the shear stress profile in Fig. 1b, $\tau (r)$ reduces to 0 at the channel centre and reaches a maximum at the channel walls of

1 $${\tau }_{w}=\frac{4\bar{U}\mu }{R}$$

Note that both $\tau (r)$ and ${\tau }_{w}$ are different to the shear stress gradient acting on the surface of a particle ($\nabla {\tau }_{p}$) flowing in a channel. While $\tau (r)$ and ${\tau }_{w}$ can be calculated as seen above, $\nabla {\tau }_{p}$ is much more complex to estimate both computationally and experimentally.

These flow regimes can be replicated experimentally in order to determine the effect they have on cell viability. The cone and plate viscometer, which is used to replicate Couette flow, has been used to apply a uniform, consistent ${\tau }_{w}$ to cells which are adherent to a plate^1^. This method, however, while useful for applying a shear, does not mimic the pipe-imposed shear that suspended cells (SCs) would ordinarily be exposed to *in vivo* as the shear gradient is constant. Continuous flow circuits, which comprise a peristaltic pump, circulating SCs around a flow circuit, have also been used. While this model more closely represents the ${\tau }_{w}$ that cells are exposed to in the CS, it fails to fully replicate its fluid dynamics^1^. The viability rates in these systems are much lower than those observed in the cone and plate experiments, with periodic exposure to ${\tau }_{w}$ of approximately 6 Pa reducing the viability of SCs down to only 20%, or even less in some cases, over 18–24 hrs^2–4^. In other cases, ${\tau }_{w}$ of 3 Pa over 24 hrs had a similar effect^5^. The third method employed in ${\tau }_{w}$ studies is the syringe and needle method, replicating Poiseuille flow. The finite volume of the syringe restricts the time duration which consequently last seconds rather than hours and so very high ${\tau }_{w}$ values were examined. It was found in several studies that, ${\tau }_{w}$ values of approximately 600–640 Pa resulted in a SC viability of 50–80% after 10 minutes^6,7^. Others have found that under lower ${\tau }_{w}$ values (2–6 Pa), viability of SCs was unaffected^8^. In both the continuous flow circuits and syringe and needle methods, due to the fact that the cells are in suspension, it is difficult to determine the actual shear that the cells were subjected to. For this reason in both of these experiments, ${\tau }_{w}$ was calculated using Eq. (1) and this was assumed to be the shear stress that the cells were exposed to in the channel. However, the cells would only experience these levels of stress if they were travelling at the wall. Therefore, from a viability perspective, it is necessary to know where the cells are located if the local fluid Poisseuille shear stress is to be estimated.

When cells or particles are transported, they can organise into distinct equilibrium locations, a phenomenon referred to as inertial migration which was first described by Segré and Silberberg in 1962^9,10^. They also gave their names to the resulting focussing effect that occurs when particles occupy equilibrium positions at 0.6 of the circular channel radius. Inertial microfluidics seeks to manipulate this focussing behaviour arising predominantly from a force balance between wall and shear-induced lift forces, but may also include rotational and deformability induced forces^11–14^. Previous studies examining these equilibrium positions of spherical particles in circular microchannels have found that at low Reynold’s numbers (Re), particles focus towards the 0.6 radius points, while an increase in the Re causes particles to focus more towards the walls^15,16^. The Re is the ratio of inertial to viscous forces in a channel and is defined as $Re=\frac{2\rho \bar{U}R}{\mu }$ where $\rho$ is the fluid density. The inertial behaviour of cells in square and rectangular microchannels has been extensively studied. Cells’ behaviour differ from those of particles as cells have a large size distribution within their population and are generally deformable. Larger cells migrate faster to equilibrium positions than their smaller counterparts^17,18^, and larger cells also migrate more in the direction of the channel centre^19,20^. Furthermore, it has been demonstrated, by both this group and others, that deformability-induced lift can cause cells with a lower Young’s Modulus to migrate towards the centre of the channel, while stiffer cells are more evenly distributed across the channel width^20–23^. Many current microfluidic devices exploit the described inertial effects due to both cell size and deformability in order to separate mixed cell solutions^21,22,24,25^. It is worth noting that all aforementioned cell migration studies, apart from the Morley *et al*. study were conducted at Re > 1.

The system design was required to be able to test a range of different conditions as cells in circulation can be subject to a wide variety of different fluidic conditions in the LS and the CS. Lymphatic capillaries are much larger (100–300 *μ*m)^26–30^ than blood capillaries (5–10 *μ*m) and fluid velocities in the LS are much lower (0.35–1 mm/s)^27–30^ than those found in the CS which can reach up to 300 mm/s. In addition, blood is a shear thinning fluid whereas lymph is considered a Newtonian fluid with a dynamic viscosity and density similar to those of water (approximately 1 mPas and 1000 kg/m^3^ respectively)^28,31,32^. Each of these individual factors combine to give lymphatic fluid flow a very low Re of approximately <1^27^. Larger capillaries and lower velocities also result in a lower ${\tau }_{w}$ in the LS. Typical ${\tau }_{w}$ values reach approximately 0.065 Pa^27,28,30^, while in the CS, they can reach 1.5–6 Pa^3^. This results in $\nabla {\tau }_{p}$ in the CS of approximately 0.004–0.023 Pa/*μ*m, however, interestingly, previous computational studies carried out in this group have found that $\nabla {\tau }_{p}$ in the LS are far in excess of this at 0.004–0.137 Pa/*μ*m. It has been postulated that these $\nabla {\tau }_{p}$ values play a large role in the metabolic response of the cells and their behaviour under certain flow conditions^33^.

**Figure 2:**
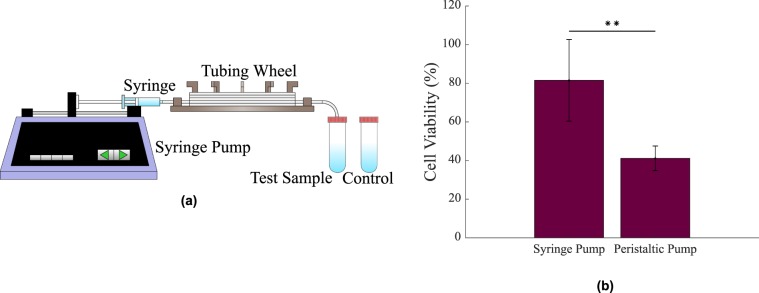
(**a**) The circulatory experimental set-up, consisting of a syringe pump infusing SCs through the *in vitro* model. (**b**) The viability of MCF-7 breast cancer cells at a ${\tau }_{w}$ value of 4 Pa in both a syringe pump and peristaltic pump. **p < 0.01, n = 3 replicates, vertical bars represent the s.e.m.

The developed system, therefore, allows for a range of vessel sizes and flow rates resulting in a large range of Re and shear stresses. It consists of microtubing surrounded by a refractive indexed matched fluid, attached to an infuse/withdraw pump. Careful consideration was given to the pump selection as it was required that shear would be imposed by the Poiseuille flow conditions alone. While a peristaltic pump may be more advantageous for replicating *in vivo* flow conditions, there were concerns that it, by its nature, may impose additional, shear forces of unknown values on the cells as they passed through it. In order to address these concerns, preliminary studies were carried out using both a peristaltic and syringe pump at flow values that exerted identical ${\tau }_{w}$ values in the tubing. As cell viability was found to be significantly higher (≈198.1%, p = 0.0042) while using the syringe pump (see Fig. 2b), it was determined that the remainder of the experiments would be conducted using this method. This delivery technique and microchannel set-up, while representative of the fluid dynamics in *ex vivo* channels, may not accurately capture the fluid dynamics that dominates *in vivo* vessels, however, given the fundamental nature of this work, the use of this approach was deemed appropriate to establish a standard for future, more in depth studies. Two representative breast cancer cell lines were assessed; MCF-7 cells and MDA-MB-231 cells. Previous studies have extensively examined the deformability or Young’s Modulus of different cancer cell lines and, more specifically, of MCF-7 cancer cells and MDA-MB-231 cancer cells. It has been noted that the more metastatic a cancer cell line is, the lower its Young’s Modulus will be. Therefore, malignant cells such as MDA-MB-231 cells possess a lower Young’s Modulus than those of benign cancer cells such as MCF-7 cells^34–37^. The viability of these cells in a cone and plate system was investigated under constant lymphatic and cardiovascular levels of ${\tau }_{w}$. The equilibrium positions of SCs under *in vivo* flow conditions were then examined. Finally, their viability in a circulatory system under $\tau (r)$ levels, calculated using these equlibrium positions, corresponding to levels of ${\tau }_{w}$ in the cone and plate was investigated to assess if $\tau (r)$ affects the viability of SCs similarly to ${\tau }_{w}$. This information will aid us in understanding circulating tumour cell migration. It will also lead to the improved design of *ex vivo* cell handling devices as the yield and viability of cells in these systems are essential.

## Results

### Viability of cancer cells in a cone and plate

**Figure 3:**
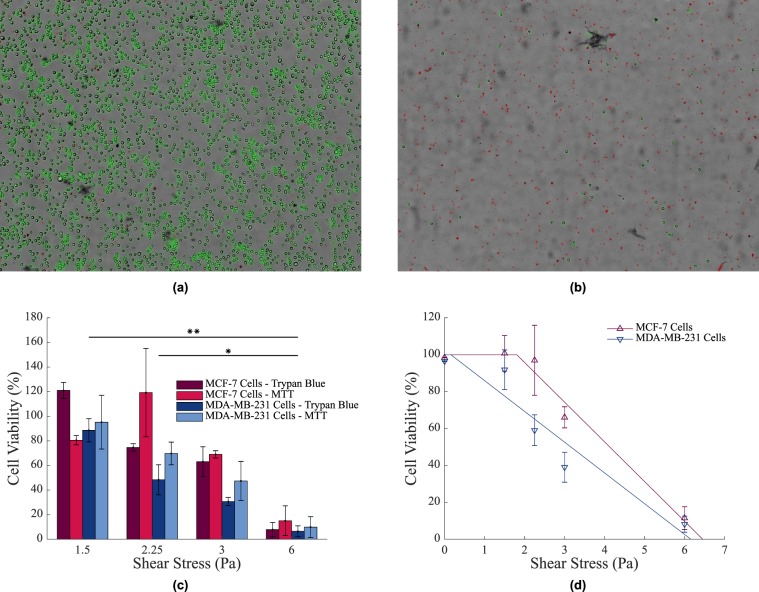
(**a**) Image of the trypan blue assay used to stain MDA-MB-231 cells to assess viability. Live cells are circled in green while dead cells have been circled in red. (**b**) Image of trypan blue stained MDA-MB-231 cells following 24 hour exposure to a shear stress of 6 Pa in a cone and plate. Cell viability has been substantially reduced from (**a**). (**c**) The viability of cells in a cone and plate as assessed by both trypan blue and MTT assays. No significant differences were recorded between the two assay types. Results were normalised to 0 Pa. **p < 0.01, *p < 0.1, n = 3 replicates, vertical bars represent the s.e.m. (**d**) The viability of cells in a cone and plate with fitted viability curve.

The viability of breast cancer cells exposed to continuous shear stress levels found within the LS and CS were investigated. Viability was assessed using both trypan blue and MTT assays. Images of the trypan blue assays taken after the cone and plate experiment was carried out (at 6 Pa) from both the control and test petri dishes are shown in Fig. 3a,b respectively. Live cells are circled in green while dead cells are circled in red.

The results of both the trypan blue and MTT assays are shown in the bar graph in Fig. 3c. There was no significant difference between results of the assay types. As was expected, an increasing continuous shear stress resulted in an increased level of cell death. For both MCF-7 and MDA-MB-231 cell lines, viability remained high (almost 100%) after being exposed to low continuous shear stress levels of 1.5 Pa for 24 hours.

A linear regression analysis was then performed on this data and a line of best fit was obtained for each cell line, indicating the viability curve with respect to increasing, continuous shear stress levels. This can be seen in Fig. 3d. As the continuous shear stress reached threshold values (approx 2 Pa for MCF-7 cells and approx 0.25 Pa for MDA-MB-231 cells), cell death began to occur. At higher continuous shear stress levels, after a period of 24 hours, only 11.5 ± 6.2% of MCF-7 cells and 8.19 ± 4.4% of MDA-MB-231 cells had survived. Besides high cell death levels, cell size decreased by approximately 70.6 ± 7.1% in MCF-7 cells and 54.9 ± 14.5% in MDA-MB-231 cells at a continuous shear rate of 6 Pa. This indicates that high continuous shear stress levels have an adverse affect on cells. Putting these values into an *in vivo* context, typical ${\tau }_{w}$ values in the LS reach 0.065 Pa^27,28,30^, while in the CS, they can reach 1.5–6 Pa^3^. Were these values to be applied in an *in vivo* scenario, it would mean that cancer cells would thrive in the LS but experience a decrease in viability in the CS. Furthermore, there is a difference between the reaction of MCF-7 cells and MDA-MB-231 cells to continuous shear stress. While no notable difference was observed between the rate of cell death with increasing continuous shear stress, there was a significant difference between the elevations of the viability curves (p = 0.01079), with MCF-7 cells surviving at greater continuous shear stress levels than MDA-MB-231 cells.

To this author’s knowledge, no previous studies utilizing a cone and plate set-up have examined the viability of MCF-7 or MDA-MB-231 cells, however previous studies have examined the effects of cone and plate induced shear on melanoma, ovarian and oesophageal cancer cells^1^. Comparison of viability rates is difficult as different sampling times and shear stress levels are used in all experiments, however, all concur with these findings; that continuous, laminar shear stress experienced by SCs can cause cell death.

### Inertial migration of cancer cells in poiseuille flow

**Figure 4:**
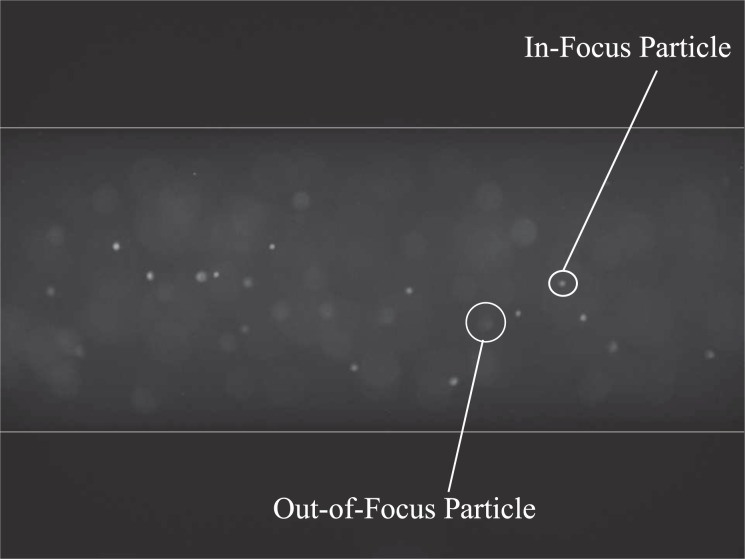
Raw image of MDA-MB-231 cells tagged with CellTrace in an 800 *μ*m microchannel, imaged using a 10x objective lens with a depth of field of 13 *μ*m and illuminated with an LED, filtered to a wavelength of 536 nm. 911 images were captured and the intensity of each pixel was averaged over the image sequence.

The inertial positions of particles and breast cancer cells in circular channels under varying flow rates, and therefore, different Re and ${\tau }_{w}$ were investigated. In order to assess the effects of cell deformability on equilibrium position, the equilibrium positions of rigid particles of 10.22 ± 0.13 *μ*m and 27–32 *μ*m diameter were also investigated. These equilibrium positions were examined at a distance of ≈20 cm from the channel inlet, resulting in a length to diameter ratio of 25–200, which is an appropriate length for inertial migration to occur^15,16,38,39^. A raw image of MCF-7 cells flowing in the microchannel is shown in Fig. 4.

**Figure 5:**
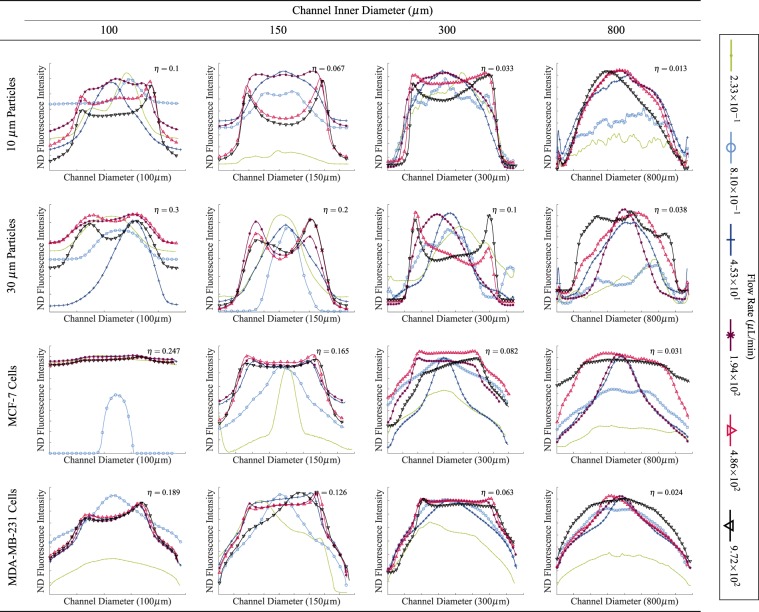
Equilibrium positions of 10 *μ*m particles, 30 *μ*m particles, MCF-7 breast cancer cells and MDA-MB-231 breast cancer cells. $\eta$ is the ratio of particle size to channel size ($\tfrac{{d}_{p}}{2R}$, where *d*_*p*_ is the particle diameter).

The intensity of the recorded fluorescence at a point in the channel is proportional to the quantity of cells or particles at that point. This intensity value was averaged over the image sequence, non-dimensionalised (ND), and plotted against the particles’/cells’ positions (see Eqs. (4) and (5)). These plots can be seen in Fig. 5.

At low flow rates (Re < 5), more typical of the LS, particles remained at the channel centre. As the flow rates increased, particles were distributed evenly across the channel diameter until finally at high flow rates (Re > 5), closer to CS values, particles obtained the inertial positions at 0.6 of the channel radius. The equilibrium positions of 10 and 30 *μ*m particles at different flow rates were found to be in good agreement with those published previously^9,10,16,40–43^. A similar phenomenon was observed in SC behaviour, however, cells failed to reach the definitive inertial positions that particles did. Instead, they were more evenly distributed across the area between the focussing points. Both the density and viscosity of DMEM with 20% Percoll are very similar to that of lymph. In addition, it has been found that lymphatic flow can be represented by Poiseuille flow^28^. Therefore, these experiments, in particular those run at fluid flow rates of 4.53 × 10^1^ *μ*L/min or less, are representative of the fluidic conditions experienced by SCs in the LS. Fluid flow rates higher than this value result in a Re typically not experienced within the lymphatics however would be representative of values found in the blood vasculature. The difference between the particle distributions and SC distributions may be attributed to either particle size or deformability. The sizes of each individual cell line vary greatly (MCF-7 cells were found to have a diameter of 24.7 ± 0.8 *μ*m while MDA-MB-231 cells had an average size of 18.9 ± 0.4 *μ*m). The range of diameter sizes was also a lot larger than the range of particle sizes which explains why cells failed to reach the definitive inertial positions that particles did.

**Figure 6:**
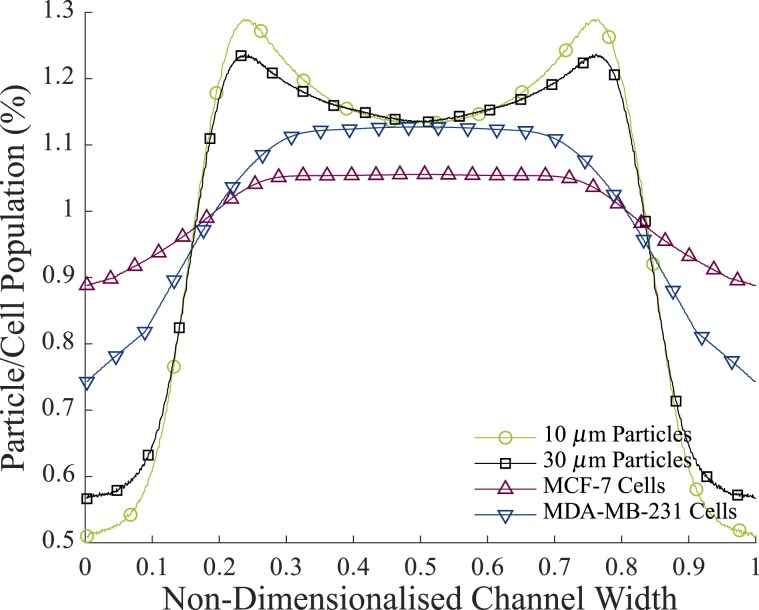
The population density distributions of cells and particles at Re > 5. The population of the particles/cells at each point in the channel are plotted as a percentage of the total particle/cell population in the channel.

Figure 6 shows the population distribution of both particles and cells across the channel width at Re > 5. Again, as is evident, both 10 and 30 *μ*m particles reached definitive inertial positions at 0.6 of the channel radius. 71.6% of 10 *μ*m particles are positioned between these two points, while 70.6% of 30 *μ*m particles are travelling in the same position. Meanwhile, MCF-7 cells and MDA-MB-231 cells were more evenly distributed throughout the channel, with MDA-MB-231 cells migrating more towards the channel centreline than MCF-7 cells. 66.3% of MDA-MB-231 cells are located between 0.6 of the channel radius, while only 62.9% of MCF-7 cells occupy the same area. This is in agreement with previous similar studies^20,21^ and it confirms that a lower Young’s Modulus causes cells to migrate towards the channel centre because the velocity gradient effects here are lower than those at the channel wall. In addition, the deformability induced lift force increases with decreasing Young’s Modulus, and so this also pushes the softer MDA-MB-231 cells towards the channel centre. Therefore the physical properties of the cells (both size and deformability) play interesting roles in the distribution of SCs in both lymphatic and vascular vessels.

### Viability of cancer cells in poiseuille flow

While cone and plates can be used to examine the cellular response to a defined shear rate, they cannot replicate the same shear experienced by these cells in a circular vessel. Additionally, circulatory experiments carried out previously, investigating the effects of shear on SC viability assume that the cells experience wall shear stress^2–8^. As is shown in Fig. 6, this assumption uses a simple parameter in order to capture a complex event. Cells do not to travel at the channel walls and so do not experience the full effect of ${\tau }_{w}$. For this reason, based off of the population distribution (Fig. 6), Poiseuille shear stress distributions and the investigated cell viability curve (Fig. 3d), a predicted cell viability curve was obtained for each cell line suspended in Poiseuille flow at varying flow rates, and therefore, varying levels of ${\tau }_{w}$. The predicted viability (*PV*) was defined as

2 $$PV={\int }_{0}^{1}\,(\frac{DP(x)}{100})(\frac{|2VC(\tau (r))|}{2})dx$$

where $DP(x)$ is the population distribution curve as defined in Eq. (6) (see Methods, Inertial Migration Experiments, Image Processing) and $VC(\tau (r))$ is the viability curve as a function of $\tau (r)$, the Poiseuille shear stress distribution. The viability curve is unique to each cell type and the Poiseuille shear stress value can be defined as

3 $$\tau (r)=({\tau }_{w})\,(2)(|x-0.5|)$$

**Figure 7:**
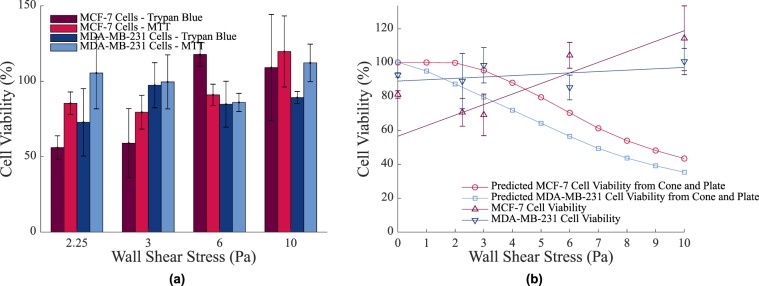
(**a**) The viability of cells in a circulation as assessed by both trypan blue and MTT assays. No significant differences were recorded between the two assay types. Results were normalised to 0 Pa. n = 3 replicates, vertical bars represent the s.e.m. (**b**) The predicted viability and experimental viability of cells in circulation. Note the values of ${\tau }_{w}$ are calculated based on the flow rate in Poiseuille Flow and are not the shear stress values experienced by the cells in the suspension.

The viability prediction curves can be seen in Fig. 7b.

The predicted viability of cell lines at varying ${\tau }_{w}$ levels in circulation is much higher than the viability levels of the same cells under the same shear stress in a cone and plate setup. For example, only 11.5 ± 6.2% of MCF-7 cells in a cone and plate at a shear rate of 6 Pa survive, however in circulation under a ${\tau }_{w}$ of 6 Pa, 70.44% of the same cells survive, based on this prediction.

The experimental viability of breast cancer cells under different flow rates under circulatory flow conditions was also investigated. The range of flow rates investigated corresponded to those values in the predicted viability model. The results of both the trypan blue and MTT assays are shown in Fig. 7a. There was no significant difference between results of the assay types. Again, a linear regression analysis was performed on this data and a line of best fit was obtained for each cell line, indicating the viability curve with respect to increasing shear stress levels. These results are also shown in Fig. 7b. In both MCF-7 cells and MDA-MB-231 cells, viability was largely unaffected by an increasing ${\tau }_{w}$ value, however a very small increase in viability was statistically significant in only the MCF-7 cells (p = 0.0019). A similar slight increase in the viability of suspended colon cancer cells with increasing ${\tau }_{w}$ over a constant time frame in a continuous flow circuit has also been previously observed^2^. At low flow rates, typical of the LS, which induced a ${\tau }_{w}$ value of 2.25 Pa (or a shear stress value of 1.35 Pa at 0.6 of the channel radius), viability in MCF-7 cells and MDA-MB-231 cells was 70.7 ± 8.2% and 89.2 ± 16.3% respectively. The MCF-7 value was 28.5% lower than predicted from the cone and plate, however the MDA-MB-231 value was close to the predicted model. An additional finding was that at a higher ${\tau }_{w}$ value, there was very little difference between the viability of the control tube and the cells in circulation. At a ${\tau }_{w}$ of 10 Pa (or a shear stress value of 6 Pa at 0.6 of the channel radius), MCF-7 and MDA-MB-231 cell viability was 2.6 times and 2.8 times larger than predicted from the cone and plate respectively. The findings suggest that inertial lift forces result in cells in circulation experiencing much lower shearing forces as they move towards the vessel centre. This raises the possibility that ${\tau }_{w}$ is not a critical force pertaining to cell viability at the flow rates experienced by cells in the LS or the CS, but rather other factors may be responsible for cell viability such as the shear stress distribution on the surface of the cells, $\nabla {\tau }_{p}$, or the shear gradient across the channel width, as has been previously suggested^33^. These findings also imply that MCF-7 cells, while more resistant to shear stress while adhered to a surface, are more sensitive to varying levels of shear and shear stress gradients whilst in suspension. Varying levels of ${\tau }_{w}$ did not impact MDA-MB-231 cells to the same extent.

These findings agree with previous studies that have examined the viability of SCs under similar ${\tau }_{w}$ values in a syringe and needle set-up, however these experiments were conducted over a much smaller time frame (30 min)^8^. This indicates that SCs have the ability to survive much longer periods in the circulation than previously thought. Additionally, it is believed that cells in a syringe and needle set-up experience high pressure points at the interface between the syringe and needle, which perhaps, affect cell viability^44^. It is interesting to note that according to these results, this may not be the case, indeed, previous studies have found that the majority of cell death occurs in the channel rather than in the delivery syringe^45^.

When interpreting the results of this study, certain limitations should be taken into consideration. While it is clear from the data that the deformability of the cell influences the inertial position and the viability of the cell, it is unclear as of yet to what extent this may be the case. Future experimental work is required in order to better quantify the influence of Young’s modulus on the inertial positions of cells. In addition, the circulatory vessel is designed on an idealised model to investigate the fundamental response of cells in Poiseuille flow. While this accurately captures the environment such cells would be exposed to in a microfluidic device, it may not accurately recapitulate the fluid mechanics of the *in vivo* vessels. Future studies in excised vessels will build on this work.

From a microfluidics viewpoint, this study furthers the limited knowledge available on the migration of deformable particles. While this area has recently seen a renewed interest, the majority of studies are computational and continue to focus on square or rectangular channels. The requirement for fundamental studies is increasing as this area continues to merge with the most common deformable particles: cells.

From a biological perspective, the results of this study have potential implications in both the areas of cancer research and lab-on-chip devices. Cancer cell metastasis is known to be a highly inefficient process with only 0.01% of circulating tumour cells actually forming tumours at secondary sites^46^. It was previously hypothesized that this low level of cancer cell extravasation was due to their inability to survive the large shearing forces in the circulatory systems^3,6,46–49^, however this study has demonstrated otherwise. Rather, it indicates that as the cells are more distributed at the channel centre, they are unlikely to travel and adhere to the vessel walls due to the fluid dynamics in a straight channel. While previous research has examined the velocity profiles of particles and red blood cells in flow bifurcations^50,51^, the inertial positions of cancer cells in such vessels would also be worth examining to investigate if cell adhesion is more likely here. More research is needed on the true factors that prevent circulating tumour cell dissemination through the circulation as these factors could potentially be harnessed in order to develop effective cancer treatments.

Finally, these findings have implications in the developing areas of lab-on-a-chip devices, flow cytometry and cell therapy. The results above indicate that cancer cells do not experience adverse effects from the shear forces imposed in a microchannel. While this is advantageous in cancer treatment, it remains to be seen if the same can be said for primary cells. These methods can be used to ascertain the threshold shear that impacts the functionality of any cell type, and therefore inform the design of any microfluidic device that may be used in the diagnosis and treatment of disease.

## Conclusion

An experimental study to investigate the inertial positions of cells and the corresponding effects of shear stress on their viability was carried out. It was found that a peristaltic pump, while ideal for replicating *in vivo* flow, imposes additional forces on cells that has a negative impact on their viability. Therefore, it is necessary to factor in this consideration when designing experiments examining effects due to the flow regime alone.

The viability response of both cells lines in a cone and plate at varying levels of shear stress were found. Upon reaching threshold values, the viability of both cell lines decreased linearly with an increase in the shear stress level. Stiffer MCF-7 cells are more resistant to this form of shear stress than MDA-MB-231 cells with approximately 30% more MCF-7 cells surviving at each shear stress level than MDA-MB-231 cells.

The distribution of 10 *μ*m particles, 30 *μ*m particles, MCF-7 cells and MDA-MB-231 cells across the channel width were found. At low Reynold’s numbers (Re < 5), cells remained at the channel centre, however as the Re increased past this, the cells became uniformly distributed between 0.6 of the channel radius. Benign, stiffer MCF-7 cells were more evenly distributed across the channel width than metastatic, deformable MDA-MB-231 cells.

Finally, the viability response of both cell lines in an *in vitro* circulatory model under varying levels of ${\tau }_{w}$ were found. Interestingly, despite the static results, the increasing shear stress rate did not significantly affect the viability of MDA-MB-231 cells and MCF-7 cells were affected only to a very slight degree. These results indicate that wall shear stress in *in vitro* conditions are not a good indicator for the viability of SCs. The local shear distribution on the cell surface may be a better indicator of cell viability in a microchannel, however, further investigations are required in order to determine if this is the case.

These results have implications in both microfluidics and cancer research. They develop the limited knowledge available on the migration of deformable particles in circular microchannels at low Reynold’s numbers. Furthermore, these methods will inform the development of future lab-on-a-chip and flow cytometry devices as well as cell therapy techniques in order to minimise damage to cells in suspension due to shear stress.

## Methods

### Cell lines and cell culture

Two representative cell lines were used in this investigation; MCF-7 breast cancer cells and MDA-MB-231 breast cancer cells (ATCC, Middlesex, UK) in order to epitomise particles of different deformabilities. MCF-7 cells were maintained in cell media, consisting of Dulbecco’s Modified Eagles Medium (DMEM), (Sigma-Aldrich Inc., Arklow, Ireland), supplemented with 10% fetal bovine serum (Sigma-Aldrich Inc., Arklow, Ireland), 1% penicillin/streptomycin (Sigma-Aldrich Inc., Arklow, Ireland), and 0.5% L-glutamine (Sigma-Aldrich Inc., Arklow, Ireland). MDA-MB-231 cells were maintained in DMEM, supplemented with 20% fetal bovine serum, 1% penicillin/streptomycin, and 1% L-glutamine. Both cell lines were cultured in an incubator at 37 °C and 5% CO_2_. Cell sizes were measured prior to experiments using a LUNA Automated Cell Counter (Logos Biosystems Inc., Villeneuve d’Ascq, France). MCF-7 cells were found to have an average diameter of 24.7 ± 0.8 *μ*m while MDA-MB-231 cells were measured to be 18.9 ± 0.4 *μ*m in diameter.

### Cone and plate experiments

#### Cell preparation

MCF-7 and MDA-MB-231 cells were cultured as described previously in petri dishes (150 mm diameter, Fisher Scientific Ireland Ltd., Dublin, Ireland). Upon reaching 70–80% confluency, cells were washed with phosphate buffered saline (PBS) and the cell culture media was replaced.

#### Experimental apparatus

Cells were placed in the cone and plate set-up, which has been described previously^52^. The entire set-up was placed in an incubator at 37 °C and 5% CO_2_. The shear stress level on the cells was gradually increased over an hour and then cells were subjected to the maximum shear stress for a further period of 23 hours. A control petri dish, which had been seeded on the same day, from the same passage, at the same cell concentration as the test petri dish, was also placed in the incubator and the viabilities of both the control and test petri dishes were assessed after 24 hours.

#### Viability acquisition and statistical analysis

Viability of cancer cells was assessed using both trypan blue and MTT assays for accuracy. Statistically, there was no significant difference between the assays. Media was aspirated off both the test and control petri dishes and reserved. The cells were washed with PBS, which was also reserved with the media, and finally, any remaining cells on both plates were detached using trypsin and added to the reserved media/PBS solutions. These solutions were centrifuged, the waste liquid was aspirated off and cells were resuspended in a small volume of fresh PBS. From these samples, the trypan blue assay was conducted. A sample of trypan blue (Sigma-Aldrich Inc., Arklow, Ireland) was mixed with a sample of the cell test suspension and control suspension. Cells were then counted and viability was measured using the LUNA Automated Cell Counter. For the MTT assay, cell samples were placed in a 96-well plate (Fisher Scientific Ireland Ltd., Dublin, Ireland) in serum-free media. MTT solution, composed of 5 mg/mL of 3-(4,5-dimethylthiazol-2-yl)-2,5-diphenyltetrazolium bromide (MTT) (Fisher Scientific Ireland Ltd., Dublin, Ireland) in PBS, was added to the cells which were then incubated for 2 hours at 37 °C and 5% CO_2_. In this time, the MTT is reduced by viable cells to purple formazan. This is then solubilized by adding a solubilisation buffer to the cell solution. The solubilisation buffer consists of 0.1 gm/mL of sodium dodecyl sulphate (SDS) (Sigma-Aldrich Inc., Arklow, Ireland) in 0.01 M of Hydrochloric Acid (HCl) (Sigma-Aldrich Inc., Arklow, Ireland). Cells were incubated again for 4 hours before the absorbance was measured at 570 nm on a spectrophotometer (Synergy H1, BioTek Instruments Inc., Swindon, UK). As absorbance is proportional to cell viability, the amount of viable cells was calculated. Each experiment was repeated at least three times and data is presented as ± standard errors from the mean. Statistical analysis was conducted using ANOVA, and two sample unequal variances were used to calculate the p-values between groups, as well as a linear regression model. All cell viability percentages are presented as percentages of the control viability.

### Inertial migration experiments

#### Microfluidic device

**Figure 8:**
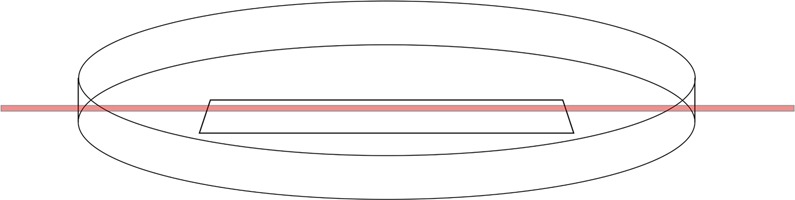
Microfluidic device.

For the inertial migration study, four different sized circular microchannels were used. These consisted of 100 *μ*m inner diameter (ID), perfluoroalkoxy alkane (PFA) tubing (Cluzeau Info Labo, Sainte-Foy-la-Grande, France), 150 *μ*m ID, PFA tubing (Cluzeau Info Labo, Sainte-Foy-la-Grande, France), 300 *μ*m ID, tetrafluoroethylene (TFE) tubing (Sigma-Aldrich Inc., Arklow, Ireland), and 800 *μ*m ID, TFE tubing (Sigma-Aldrich Inc., Arklow, Ireland). These sizes were chosen as they most accurately represent the range of diameters that human lymphatic vessels typically grow to. A schematic of the microfluidic device is shown in Fig. 8. Petri dishes (90 mm diameter, Fisher Scientific Ireland Ltd., Dublin, Ireland) had holes drilled at both ends and an 18 × 44 mm window was cut into the base of the dish. A glass slide was attached over this window. Tubing was cut to a length of approximately 40 cm, threaded through the holes in the petri dish and attached. As water has a similar refractive index (1.33) to both PFA (1.34) and TFE (1.35), the petri dish was filled with water in order to prevent optical distortion during image acquisition. Similar devices have been used previously^53,54^.

#### Particle suspension

In order to assess the effects of cell deformability on equilibrium position, the equilibrium positions of rigid particles were also investigated. Fluorescent microparticles of 10.22 ± 0.13 *μ*m diameter (microParticles GmbH, Berlin, Germany), and 27–32 *μ*m diameter (Cospheric LLC, California, USA) were used. These particle sizes represent the range of cell sizes that the two breast cancer cell lines grow to^20^. The particles were mixed at a 0.08% weight fraction in distilled water, with 1% volume fraction of Tween-20 surfactant (Sigma-Aldrich Inc., Arklow, Ireland), and a percentage of glycerol (Sigma-Aldrich Inc., Arklow, Ireland) (22% for the 10.22 *μ*m particles and 34% for 27–32 *μ*m particles). The Tween-20 prevents particle aggregation while the glycerol matches the density of the water to that of the particles. The dynamic viscosity and density of distilled water are 1 mPas and 1000 kg/m^3^ respectively.

#### Cell suspension

MCF-7 and MDA-MB-231 cells were cultured as described previously. Upon reaching 70–80% confluency, cells were detached using trypsin (Sigma-Aldrich Inc., Arklow, Ireland) and fluorescently labelled using CellTrace Yellow (Bio-Sciences, Dublin, Ireland). They were then resuspended at a concentration of approximately 750 cells/*μ*L, in serum-free DMEM and 20% Percoll (Sigma-Aldrich Inc., Arklow, Ireland). Percoll prevents cell settling and adhesion and has no effect on cell viability^55^. This concentration of cells was used for visualization purposes and is not representative of the concentration of circulating tumour cells in the CS (≈1–10 cells/mL^56^). The concentration of circulating tumour cells in the LS is currently unknown. The dynamic viscosity and density of DMEM with 20% Percoll are 1.17 mPas and 1000 kg/m^3^ respectively.

#### Experimental apparatus

**Figure 9:**
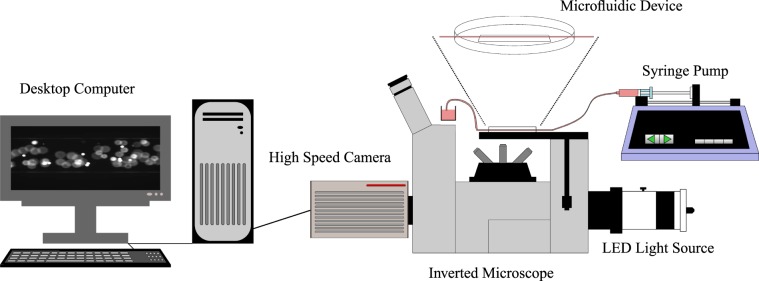
The inertial migration experimental set-up, consisting of LED light source, connected to a microscope and high-speed camera.

Figure 9 displays the experimental set-up used in the inertial migration experiments. A syringe pump (Pump 11 Elite, Harvard Apparatus, Cambourne, UK) was used to infuse the solution through the *in vitro* model at a constant flow rate. A range of flow rates were investigated, which are outlined, along with their corresponding Re, in Table 1a. An inverted microscope (IX73, Olympus, Southend-on-Sea, UK), was focussed at the channel centre, at a distance of ≈20 cm from the channel inlet. This distance corresponded to a channel length to channel diameter ratio $(\tfrac{L}{2R})$ of 25–200. Previous computational and experimental work has found that at lower Re (see Table 1a), the entry length for rigid particles is smaller, therefore, it is believed that this channel length was sufficient in order to achieve fully developed radial migration^15,16,39^. Previous studies examining the migration length of deformable red blood cells have shown that in similarly-sized microchannels, a migration length of ≈20 mm is a sufficient length in order to achieve fully developed radial migration^38^. As cancer cells are considered to be less deformable than red blood cells, and the channel length is sufficient for the inertial migration of both rigid particles and deformable red blood cells, it is believed to be of adequate length to achieve the inertial migration of deformable cancer cells. Different objective lenses were used, depending on the channel size, these are outlined in Table 2. A white LED light source (pE-100, CoolLED Ltd., Andover, UK), shining through a filter cube (Excitation: 536/40 nm, Emission: 607/36 nm) was used to illuminate the fluorescent particles (40% light intensity) or cells (100% light intensity) as they passed through the microfluidic device. A high-speed camera (Orca Flash 2.8, Hamamatsu Photonics K.K., Welwyn Garden City, UK), was used to capture the images which were relayed to the computer.

**Table 1:** (**a**) Channel IDs, investigated flow rates and corresponding Re used in the inertial migration experiments.

(a)					
		Channel Inner Diameter (*μ*m)			
		100	150	300	800
Flow Rate (*μ*L/min)	2.33 × 10^−1^	0.042	0.028	0.014	0.005
	8.1 × 10^−1^	0.147	0.098	0.049	0.018
	4.53 × 10^1^	8.22	5.48	2.74	1.03
	1.94 × 10^2^	35.15	23.43	11.72	4.39
	4.86 × 10^2^	88.2	58.8	29.4	11.0
	9.72 × 10^2^	175.8	117.2	58.6	22.0
(**b**)					
**Flow Rates** (***μ*L**/**min**)				***τ***_***w***_ (**Pa**)	**Re**
1.13 × 10^1^				2.25	2.05
1.51 × 10^1^				3	2.74
3.01 × 10^1^				6	5.48
5.03 × 10^1^				10	9.13

(**b**) Investigated flow rates and corresponding ${\tau }_{w}$ and Re values used in circulatory experiments.

**Table 2:** Experimental Objective Lenses.

Tubing ID (*μ*m)	Magnification	Numerical Aperture	Depth of Field
100	40x	0.65	1 *μ*m
150	20x	0.50	3 *μ*m
300			
800	10x	0.30	12 *μ*m

#### Image acquisition

Images were captured using the software HCImageLive (Hamamatsu Photonics K.K., Welwyn Garden City, UK). For each inertial migration experiment, a series of 911, 8 bit, greyscale images with a resolution of 960 × 720 pixels were captured. A gain of 0–50 was used for particles while a gain of 200–255 was used for cells, which are less fluorescent. The exposure time for each image was 20 ms and each image sequence was collected over a period of 20 s.

#### Image processing

Image sequences were processed using a MATLAB script (MathWorks, Galway, Ireland). The tubing wall was located and all images were cropped to this size. An image matrix was then calculated by averaging the intensity values of the pixels

4 $$IAv=\frac{{\sum }_{m=1}^{911}\,{I}_{m}}{911}$$

where $IAv$ is an image matrix of the average intensity values for each pixel, averaged over the image sequence. ${I}_{m}$ is the *m*^*th*^ image in the image sequence. Following this, the intensity distribution function was calculated

5 $$DI(x)=\frac{{\sum }_{n=1}^{L}\,{(IAv)}_{x,n}}{L}$$

where $DI(x)$ is the intensity distribution as a function of $x$, the position across the channel diameter and $L$ is the length of the image in pixels. Finally, the population distribution curves were calculated using

6 $$DP(x)=\frac{DI(x)}{{\int }_{0}^{1}\,DI(x)dx}$$

where $DP(x)$ is the population distribution curve as a function of $x$. These distribution curves were averaged for each particle/cell type in order to obtain population density plots.

### Circulatory experiments

#### Cell suspension

MCF-7 and MDA-MB-231 cells were cultured as described previously. Upon reaching 70–80% confluency, cells were detached and resuspended at a concentration of approximately 750 cells/*μ*L, in serum-free DMEM and 20% Percoll.

#### Experimental apparatus

Figure 2a displays the experimental set-up used in the circulatory experiments. A syringe pump was used to infuse and withdraw the cell suspension through the *in vitro* model at a constant flow rate. The viability of MCF-7 cells under a ${\tau }_{w}$ value of 4 Pa was compared between a peristaltic pump and a syringe pump (see Fig. 2b). The inner diameter of the tubing in the syringe pump was 500 *μ*m and the estimated change in the inner diameter of the tubing was calculated to be ≈ −280 *μ*m. This resulted in an increased ${\tau }_{w}$ value at the pinched section of ≈9 Pa. As viability in the syringe pump was significantly higher than that in the peristaltic pump (p = 0.0042), the syringe pump was chosen as the method of delivery. The *in vitro* model consisted of flexible tubing (ID: 100 *μ*m as described previously) of ≈30 cm in length and different flow rates were also used (See Table 1b). Fluid was delivered through a circular syringe of 14.5 mm in diameter. It has previously been found that lymphatic flow can be accurately represented by Poiseuille flow^28^. A cell suspension volume of approximately 2 mL was placed in the syringe. When this volume was completely infused into the previously empty test sample tube, the syringe pump was programmed to withdraw the solution. Cells were infused and withdrawn continuously over a period of 24 hours. Due to the small volume of fluid that was being passed through the tubing, the time that the cells were not in the tubing was deemed negligible when estimating the viability of the cells in the tubing. The entire set-up was placed in an incubator at 37 °C and 5% CO_2_ for this time. A vial of suspended cells, acting as the control, was also placed in the incubator. Following this, both the control and test samples were assessed for viability.

#### Viability acquisition and statistical analysis

Viability of SCs was assessed using both trypan blue and MTT assays for accuracy, using the procedures which have been described previously. Statistically, there was no significant difference between the assays. Each experiment was repeated at least three times and data is presented as ± standard errors from the mean. Statistical analysis was conducted using ANOVA, and two sample unequal variances were used to calculate the p-values between groups. All cell viability percentages are presented as percentages of the control viability.

## Data Availability

The datasets generated during and/or analysed during the current study are available from the corresponding author on reasonable request.

## References

[1] Krog, B. L. & Henry, M. D. *Biomechanics of the Circulating Tumor Cell Microenvironment*, vol. 1092 of *Advances in Experimental Medicine and Biology*, 209–233 (Springer International Publishing Ag, Cham, 2018). 10.1007/978-3-319-95294-9_11 PMC7304329 30368755

[2] Fan R (2016). Circulatory shear flow alters the viability and proliferation of circulating colon cancer cells. Scientific Reports.

[3] Regmi S, Fu A, Luo KQ (2017). High shear stresses under exercise condition destroy circulating tumor cells in a microfluidic system. Scientific Reports.

[4] Jin J, Tang K, Xin Y, Zhang TL, Tan YH (2018). Hemodynamic shear flow regulates biophysical characteristics and functions of circulating breast tumor cells reminiscent of brain metastasis. Soft Matter.

[5] Fu A (2016). High expression of mnsod promotes survival of circulating breast cancer cells and increases their resistance to doxorubicin. Oncotarget.

[6] Barnes JM, Nauseef JT, Henry MD (2012). Resistance to fluid shear stress is a conserved biophysical property of malignant cells. Plos One.

[7] Mitchell MJ (2015). Lamin a/c deficiency reduces circulating tumor cell resistance to fluid shear stress. American Journal of Physiology-Cell Physiology.

[8] Triantafillu UL, Park S, Klaassen NL, Raddatz AD, Kim Y (2017). Fluid shear stress induces cancer stem cell-like phenotype in mcf7 breast cancer cell line without inducing epithelial to mesenchymal transition. International Journal of Oncology.

[9] Segre G, Silberberg A (1962). Behaviour of macroscopic rigid spheres in poiseuille flow.1. Determination of local concentration by statistical analysis of particle passages through crossed light beams. Journal of Fluid Mechanics.

[10] Segre G, Silberberg A (1962). Behaviour of macroscopic rigid spheres in poiseuille flow.2. experimental results and interpretation. Journal of Fluid Mechanics.

[11] Zhang J (2016). Fundamentals and applications of inertial microfluidics: a review. Lab on a Chip.

[12] Morley ST, Walsh MT, Newport DT (2017). Opportunities for studying the hydrodynamic context for breast cancer cell spread through lymph flow. Lymphatic Research and Biology.

[13] Gou YX, Jia YX, Wang P, Sun CK (2018). Progress of inertial microfluidics in principle and application. Sensors.

[14] Stoecklein D, Di Carlo D (2019). Nonlinear microfluidics. Analytical Chemistry.

[15] Asmolov ES (1999). The inertial lift on a spherical particle in a plane poiseuille flow at large channel reynolds number. Journal of Fluid Mechanics.

[16] Matas JP, Morris JF, Guazzelli E (2004). Inertial migration of rigid spherical particles in poiseuille flow. Journal of Fluid Mechanics.

[17] Tanaka T (2012). Inertial migration of cancer cells in blood flow in microchannels. Biomedical Microdevices.

[18] Kulasinghe A, Zhou J, Kenny L, Papautsky I, Punyadeera C (2019). Capture of circulating tumour cell clusters using straight microfluidic chips. Cancers.

[19] Lim EJ, Ober TJ, Edd JF, McKinley GH, Toner M (2012). Visualization of microscale particle focusing in diluted and whole blood using particle trajectory analysis. Lab on a Chip.

[20] Morley ST, Walsh MT, Newport DT (2017). The advection of microparticles, mcf-7 and mda-mb-231 breast cancer cells in response to very low reynolds numbers. Biomicrofluidics.

[21] Hur SC, Henderson-MacLennan NK, McCabe ERB, Di Carlo D (2011). Deformability-based cell classification and enrichment using inertial microfluidics. Lab on a Chip.

[22] Hur SC, Mach AJ, Di Carlo D (2011). High-throughput size-based rare cell enrichment using microscale vortices. Biomicrofluidics.

[23] Villone MM, Maffettone PL (2019). Dynamics, rheology, and applications of elastic deformable particle suspensions: a review. Rheologica Acta.

[24] Takagi J, Yamada M, Yasuda M, Seki M (2005). Continuous particle separation in a microchannel having asymmetrically arranged multiple branches. Lab on a Chip.

[25] Sun JS (2013). Size-based hydrodynamic rare tumor cell separation in curved microfluidic channels. Biomicrofluidics.

[26] Pan WR, Le Roux CM, Levy SM, Briggs CA (2010). The morphology of the human lymphatic vessels in the head and neck. Clinical Anatomy.

[27] Dixon JB (2006). Lymph flow, shear stress, and lymphocyte velocity in rat mesenteric prenodal lymphatics. Microcirculation.

[28] Rahbar E, Moore JE (2011). A model of a radially expanding and contracting lymphangion. Journal of Biomechanics.

[29] Margaris KN, Nepiyushchikh Z, Zawieja DC, Moore J, Black RA (2016). Microparticle image velocimetry approach to flow measurements in isolated contracting lymphatic vessels. Journal of Biomedical Optics.

[30] Kornuta JA (2015). Effects of dynamic shear and transmural pressure on wall shear stress sensitivity in collecting lymphatic vessels. American Journal of Physiology-Regulatory Integrative and Comparative Physiology.

[31] Jafarnejad M, Woodruff MC, Zawieja DC, Carroll MC, Moore JE (2015). Modeling lymph flow and fluid exchange with blood vessels in lymph nodes. Lymphatic Research and Biology.

[32] Cooper LJ, Heppell JP, Clough GF, Ganapathisubramani B, Roose T (2016). An image-based model of fluid flow through lymph nodes. Bulletin of Mathematical Biology.

[33] Morley ST, Newport DT, Walsh MT (2017). Towards the prediction of flow-induced shear stress distributions experienced by breast cancer cells in the lymphatics. Biomechanics and Modeling in Mechanobiology.

[34] Lee MH (2012). Mismatch in mechanical and adhesive properties induces pulsating cancer cell migration in epithelial monolayer. Biophysical Journal.

[35] Corbin EA, Kong F, Lim CT, King WP, Bashir R (2015). Biophysical properties of human breast cancer cells measured using silicon mems resonators and atomic force microscopy. Lab on a Chip.

[36] Coceano G (2016). Investigation into local cell mechanics by atomic force microscopy mapping and optical tweezer vertical indentation. Nanotechnology.

[37] Yousafzai MS (2017). Investigating the effect of cell substrate on cancer cell stiffness by optical tweezers. Journal of Biomechanics.

[38] Noso R, Kimura T, Sakamoto K, Sugihara-Seki M, Seki J (2015). Cross-sectional distributions of platelet-sized particles in blood flow through microchannels. Nihon Reoroji Gakkaishi.

[39] Nakayama S, Yamashita H, Yabu T, Itano T, Sugihara-Seki M (2019). Three regimes of inertial focusing for spherical particles suspended in circular tube flows. Journal of Fluid Mechanics.

[40] Kim YW, Yoo JY (2008). The lateral migration of neutrally-buoyant spheres transported through square microchannels. Journal of Micromechanics and Microengineering.

[41] Choi YS, Lee SJ (2010). Holographic analysis of three-dimensional inertial migration of spherical particles in micro-scale pipe flow. Microfluidics and Nanofluidics.

[42] Kim YW, Yoo JY (2010). Two-phase flow laden with spherical particles in a microcapillary. International Journal of Multiphase Flow.

[43] Kim YW, Noh H, Jin S, Yoo JY (2011). Inertial-microfluidic radial migration in solid/liquid two-phase flow through a microcapillary: Particle equilibrium position. Experiments in Fluids.

[44] Wahlberg B, Ghuman H, Liu JR, Modo M (2018). *Ex vivo* biomechanical characterization of syringe-needle ejections for intracerebral cell delivery. Scientific Reports.

[45] Suwannaphan, T. *et al*. Investigation of leukocyte viability in a setup of spiral microchannel for cell sorting application. In *11th Biomedical Engineering International Conference (BMEiCON)*, Biomedical Engineering International Conference (Ieee, New York, 2018).

[46] Chiang SPH, Cabrera RM, Segall JE (2016). Tumor cell intravasation. American Journal of Physiology-Cell Physiology.

[47] Azevedo AS, Follain G, Patthabhiraman S, Harlepp S, Goetz JG (2015). Metastasis of circulating tumor cells: Favorable soil or suitable biomechanics, or both?. Cell Adhesion & Migration.

[48] Goetz JG (2018). Metastases go with the flow. Science.

[49] Xin Y (2019). Mechanics and actomyosin-dependent survival/chemoresistance of suspended tumor cells in shear flow. Biophysical Journal.

[50] Doutel E, Pinto SIS, Campos J, Miranda JM (2013). mu piv analysis and numerical simulation of the flow in mili-scale channels developed for studies in hemodynamics. 3rd International Conference on Biomedical Engineering and Technology - Icbet 2013.

[51] Kodama Y, Aoki H, Yamagata Y, Tsubota K (2019). *In vitro* analysis of blood flow in a microvascular network with realistic geometry. Journal of Biomechanics.

[52] Franzoni M (2016). Design of a cone-and-plate device for controlled realistic shear stress stimulation on endothelial cell monolayers. Cytotechnology.

[53] Chiavaroli S, Newport D, Woulfe B (2010). An optical counting technique with vertical hydrodynamic focusing for biological cells. Biomicrofluidics.

[54] Buchanan CF (2014). Three-dimensional microfluidic collagen hydrogels for investigating flow-mediated tumor-endothelial signaling and vascular organization. Tissue Engineering Part C-Methods.

[55] Pertoft H (2000). Fractionation of cells and subcellular particles with percoll. Journal of Biochemical and Biophysical Methods.

[56] Hou HW (2013). Isolation and retrieval of circulating tumor cells using centrifugal forces. Scientific Reports.

