# Author Correction: The *in vitro* inertial positions and viability of cells in suspension under different *in vivo* flow conditions

**DOI:** 10.1038/s41598-020-66681-8

**Published:** 2020-06-12

**Authors:** Sinead Connolly, Kieran McGourty, David Newport

**Affiliations:** 1 School of Engineering, Bernal Institute, University of Limerick, Limerick, Ireland; 2 School of Natural Sciences, Bernal Institute, Health Research Institute, University of Limerick, Limerick, Ireland

**Correction to:** *Scientific Reports* 10.1038/s41598-020-58161-w, published online 03 February 2020

The original version of this Article contained an error in Affiliation 2, which was incorrectly given as ‘School of Natural Sciences, Bernal Institute, University of Limerick, Limerick, Ireland’. The correct affiliation is listed below:

School of Natural Sciences, Bernal Institute, Health Research Institute, University of Limerick, Limerick, Ireland

This error has now been corrected in the HTML and PDF versions of the Article.

